# Signal transduction in non-climacteric fruit ripening

**DOI:** 10.1093/hr/uhac190

**Published:** 2022-08-25

**Authors:** Wei Wang, Dingyu Fan, Qing Hao, Wensuo Jia

**Affiliations:** College of Horticulture, China Agricultural University, Beijing 100193, China; Institute of Horticulture Crops, Xinjiang Academy of Agricultural Science, Urumqi 830091, Xinjiang, China; Institute of Horticulture Crops, Xinjiang Academy of Agricultural Science, Urumqi 830091, Xinjiang, China; College of Horticulture, China Agricultural University, Beijing 100193, China

## Abstract

Fleshy fruit ripening involves changes in numerous cellular processes and metabolic pathways, resulting from the coordinated actions of diverse classes of structural and regulatory proteins. These include enzymes, transporters and complex signal transduction systems. Many aspects of the signaling machinery that orchestrates the ripening of climacteric fruits, such as tomato (*Solanum lycopersicum*), have been elucidated, but less is known about analogous processes in non-climacteric fruits. The latter include strawberry (*Fragaria* x ananassa) and grape (*Vitis vinifera*), both of which are used as non-climacteric fruit experimental model systems, although they originate from different organs: the grape berry is a true fruit derived from the ovary, while strawberry is an accessory fruit that is derived from the floral receptacle. In this article, we summarize insights into the signal transduction events involved in strawberry and grape berry ripening. We highlight the mechanisms underlying non-climacteric fruit ripening, the multiple primary signals and their integrated action, individual signaling components, pathways and their crosstalk, as well as the associated transcription factors and their signaling output.

## Introduction

The ripening of fleshy fruits involves major physiological, structural, and metabolic changes, resulting in alterations in color, sugar levels, acidity, texture, and aroma. These changes render fruits edible and desirable to animals, there enhancing seed dispersal, and are fundamental to their value as agricultural commodities [1, 2]. Indeed, fruit ripening is tightly associated with its quality, and so understanding its regulation by both development-initiated and environmentally derived signals is of great significance for commercial fruit production. Fleshy fruits are physiologically categorized into the climacteric (CL) or non-climacteric (NC) categories: CL fruits undergo a burst of CO_2_ and ethylene production at the onset of ripening while NC fruits do not. Mechanistic studies of fruit ripening have typically focused more on CL fruits, with tomato (*Solanum lycopersicum*) representing the most studied model [1, 3–7]. In recent years, however, there has been growing interest in molecular studies of NC fruit ripening, particularly using strawberry (*Fragaria*$\times$ ananassa) as a model [8–11].

It is well established that coordinated organ growth and development is achieved via cellular signal transduction, spanning primary signal perception triggered by internal and external cues, to the responses affecting a cascade of downstream cellular metabolic events. Plant hormones play a pivotal role in the regulation of fruit ripening and the ripening of CL fruits is predominantly induced by the gaseous hormone ethylene, whereas there is increasing evidence that the regulation of NC fruit ripening involve multiples hormones. Both CL and NC fruit ripening are affected by environmental factors, including light, temperature, and water status. The effect of these factors on ripening may be imposed via independent signaling systems or through a modification of the hormone signaling pathways. In prokaryotic cells, signal transduction from primary signal perception to an output can be achieved by two-component systems in which a response regulator acts to regulate gene expression [12], whereas in eukaryote cells the signaling mechanisms are far more complex, involving different signal components as well as transcription factors (TFs) that act to mediate the signaling output in a signaling cascade.

Over the past decades, ethylene signal transduction has been extensively studied and the main signal components and cascades have been identified and characterized in the experimental model plant *Arabidopsis thaliana*. Using information from Arabidopsis research as a template, studies to elucidate CL fruit ripening-associated signal transduction have progressed rapidly, particularly in tomato [3–7, 13–16]. In contrast, relatively little is known about NC fruit ripening associated-signal transduction, although progress has been made in recent years in elucidating NC fruit ripening with strawberry emerging as a model plant. An overall picture of the signaling network controlling NC fruit ripening is emerging. Given that strawberry and the grape berry are both NC model fruits, but are derived from different organs, in this article we review recent insights into fruit ripening signal transduction in both fruits. We highlight the mechanisms associated with the origin of multiple primary signals and their coordinated action, the signaling components, pathways and their crosstalk, as well as the TFs and their signaling output.

### Early signal production regulating changes in fruit physiology and structure

In addition to well characterized fruit quality-associated parameters that are linked with ripening, such as color, texture, and aroma, another typical event is a large decrease in cellular osmotic potential. This results from the accumulation of the soluble solids, which are primarily sugars and organic acids. However, little attention has been paid in the literature to the significance of the osmotic potential-associated changes in the regulation of fruit ripening. Such an effect was recently shown in a study by Jia *et al.* [17] where a sharp decline in cellular osmotic potential coincided with the onset of strawberry fruit ripening. Moreover, by manipulating the changes in osmotic potential, the authors reported being able to modulate ripening. As discussed below, the phytohormone abscisic acid (ABA) is regarded as a key signal controlling NC fruit ripening. Given that ABA accumulation can be induced by various environmental stresses, notably dehydration and osmotic stresses [18, 19], it follows that fruit ripening-associated ABA accumulation may be derived from the decrease in osmotic potential, or that the decreased osmotic potential may trigger the origin of the ABA signal.

To better understand the structural basis for NC fruit ripening, in a recent study we examined patterns of anatomical changes in strawberry fruit from anthesis to ripening [20]. The interval from strawberry fruit anthesis to the onset of ripening is approximately 4 weeks and it can be divided into five major stages: small green, middle green, large green, white and the reddening stage. We observed that cell separation occurred during anthesis, and a clear separation could be observed only one week after anthesis. Strikingly, full separation coincides with the initiation of an overall degradation of the cell walls, which again coincides with the onset of a series of fruit ripening-associated physiological changes.

The observation that cell separation initiated at the very early stage of fruit development (i.e. during anthesis) raises the question of whether cell separation may represent a very early signal controlling fruit ripening. It has been well established that the cell wall status is intimately associated with cell signal transduction, due to the many wall-associated receptors, protein kinases, and peptide signals in the wall and the wall-plasma membrane interface [21–24]. Moreover, some wall metabolites may be able to act directly as signals involved in the regulation of plant growth and development or stress responses [23, 25–27]. In addition, cell walls can be thought of as providing a pool of calcium and to play a crucial role in calcium signaling, which has been associated with fruit ripening [28–36]. Given the importance of the cell wall in cellular signal transduction, it seems likely that wall degradation could have profound impacts on fruit ripening-related signaling. Furthermore, because cell wall degradation is associated with cell separation, it is reasonable to propose that cell separation may serve as an early signal in fruit ripening regulation.

Cell separation results from the degradation of the middle lamella, which is well known to be mainly comprised of pectin (polygalacturonic acid). Polygalacturonase (PG) has been well demonstrated to be a key enzyme catalyzing pectin degradation. In the past years, PG has been extensively studied owing to its pivotal role in the regulation of fruit firmness [3, 8, 15, 30, 37]. From a point of view of signal transduction, there should exist a signaling cascade that mediates the transcriptional or post-transcriptional regulation of PG activity, but to date, little is known about the signaling cascade upstream of the PG regulation. Because PG-catalyzed cell separation is proposed to be linked to the early signaling, identification of the TFs controlling the PG gene expression is undoubtedly of great significance for profoundly deciphering the signaling mechanisms behind NC fruit ripening.

### Hormonal signals involved in the regulation of fruit ripening

While ethylene is well established as the primary signal controlling the ripening of CL fruit, the signaling system that modulates NC fruit ripening is still not well resolved and multiple plant hormones have been proposed to be involved. Early studies of the effect of hormones on NC fruit ripening largely involved pharmacological experiments and measurements of hormone levels during fruit development and ripening. For example, the application of cytokinin, indole acetic acid (IAA, an auxin), and gibberellin were reported to promote growth of the NC fruit, fig (*Ficus carica L*) [37] and auxin levels were found to increase during citrus (*Citrus reticulata Blanco*) fruit development [38]. In a classic study, Given *et al.* [39] found that removal of achenes from strawberry fruit promoted pigmentation, and that application of the synthetic auxins to the de-achened fruit surface delayed pigmentation. These observations suggested that auxin is an important factor in controlling strawberry fruit ripening. It was later reported that FaAux/IAA1/2, two TFs from the auxin signaling pathway, are involved in early strawberry fruit growth and development [40], and another study found that the expression of some members of the FaAux/IAA and FaARF families (auxin response factors) increased with the onset of receptacle ripening. Taken together, these studies lead to the conclusion that IAA is a key signal controlling strawberry fruit development and ripening. Similarly, several studies have demonstrated that application of auxin or NAA (the synthetic auxin, 1-naphthaleneacetic acid) delays grape berry ripening [41–45]. More recently, the global pattern of the auxin-induced gene expression in grape berry was reported, in which it was found that application of NAA to pre-veraison grape berries resulted in a significant change in the expression of many genes [46]. Among these, the expression of many genes encoding putative cell wall catabolic enzymes significantly decreased, while the expression of those encoding putative cellulose synthases increased. Accordingly, it was concluded that auxin treatment of grape berries delays ripening onset by inhibiting cell wall metabolism. Particular attention has been paid to the role of ABA in the regulation of NC fruit ripening [19]. An early study reported that the expression of several genes encoding key proteins in the ABA signaling pathway, such *ABI1* and *ABI2*, correlated with strawberry fruit development and ripening [47]. Later studies also suggested a role for ABA in the regulation of strawberry fruit ripening. For example, it was reported that as ripening proceeds, ABA levels and the gene expression of 9-*cis*-epoxycarotenoid dioxygenase (NCED), a key enzyme in ABA biosynthesis pathway, progressively increase and peak at the fully ripe stage [48–51]. An analysis of the expression profiles of ABA responsive genes during strawberry receptacle fruit ripening revealed that many TFs, such as the WRKY-heat shock factor (HSF) and KNOTTED1-like homeobox, were differently regulated in response to ABA treatment [52–55].

More direct evidence supporting the involvement of ABA in strawberry fruit ripening was provided by several studies using virus-induced gene silencing (VIGS) [51, 56, 57], where it was reported that VIGS-induced down-regulation of *FaNCED1*, a key gene in the ABA synthesis pathway, or *FaCHLH/ABAR*, a putative ABA receptor, arrested ripening. However, it is has been suggested that the VIGS in strawberry can inherently perturb ripening [58], so additional studies using other approaches to silence gene expression may be valuable.

There is no doubt that ABA is an important regulator of NC fruit ripening, as even for CL fruits, ABA has been increasingly suggested to be an important regulator of fruit ripening [59]. However, it cannot be ignored that there is evidence that exogenous application of ABA was not able to significantly promote strawberry fruit ripening [48]. More recently, we examined the effect of exogenous ABA on strawberry fruit ripening and found that it might depend on the method of application: feeding ABA via the fruit pedicel did indeed induce ripening, whereas injection directly into the receptacle did not have a significant effect. To investigate this phenomenon further, we examined the pattern of ABA accumulation when ABA was fed via the fruit stalk, and found that the ABA fed was mainly accumulated in the achenes, but not the receptacle. Accordingly, we concluded that it is the achenes, rather than the receptacle that are regulated by ABA. We further provided evidence that the effect of ABA on receptacle ripening likely occurs via a modification of IAA transport from the achenes to the receptacle [60].

In grape, evidence of a role for ABA in fruit ripening has mainly resulted from pharmacological experiments. Application of exogenous ABA was reported to induce berry ripening, as indicated by accumulation of fruit ripening-associated metabolic products, such as anthocyanins, flavonols, resveratrol and others [61–64]. Furthermore, the expression of several ABA biosynthesis and signaling-related genes was found to increase at the onset of grape berry ripening [65]. In another study, over-expression of *VvABF2*, which encodes an ABA response element-binding factor, in transgenic grape cell suspensions resulted in an accumulation of stilbenes, while heterologous expression of *VvABF2* in tomato caused a decrease in fruit firmness [66]. Collectively, these observations suggest that ABA contributes to the regulation of grape berry ripening. In sweet cherries, endogenous concentration of ABA was reported to be increased progressively during fruit growth and ripening on the tree, which was positively correlated with anthocyanin and vitamin E accumulations during pre-harvest, implying an important role of ABA in the regulation of sweet cherry fruit ripening and quality formation [67].

In addition to IAA and ABA, there is evidence that jasmonic acid (JA) may also be involved in regulating NC fruit ripening. In strawberry, several studies have demonstrated that application of exogenous methyl jasmonate (MeJA) significantly promoted the accumulation of ripening-associated compounds, such as anthocyanins [68–72], as well as the production of aroma compounds [73]. Another study reported that exogenous MeJA application caused a decrease in ABA content, and it was proposed that MeJA might act antagonistically with ABA in the regulation of strawberry fruit ripening. Moreover, it was shown that exogenous MeJA altered the expression profile of several cell wall metabolism-associated genes [69]. Interestingly, it was also found that the expression of all *JAZ* genes, encoding a key signaling component in the JA signaling pathway, substantially decreased during strawberry fruit development and ripening [71]. Because JAZ proteins act to arrest the activity of MYC2, the core signal in the JA signaling pathway, the decrease in *JAZ* expression suggests that JA promotes strawberry fruit ripening [70]. Similarly, in grape berry, a number of pharmacological studies have demonstrated that application of MeJA promotes the accumulation of a variety of ripening-associated compounds, such as anthocyanins [74, 75], phenolic compounds [76–78], resveratrol [79], and volatile compounds [69].

More recently, Alferez *et al.* (2021) reviewed the interplay between ABA and Gibberellins as related to ethylene in NC fruit ripening [11]. It was proposed that there exists a competition for the metabolic precursor geranylgeranyl pyrophosphate (GGPP) between GA and ABA biosynthesis, such that the decrease in GA biosynthesis before fruit ripening may contribute to the ABA accumulation during NC fruit ripening. Moreover, it was proposed that ABA may act to promote the sensitivity to ethylene, and therefore, ethylene may play a role in NC fruit ripening owing to the ABA-induced increase in the ethylene sensitivity, regardless of an increase in ethylene production. In support of such a proposal, a study by Tosetti *et al.* (2020) reported that continuous exposure to ethylene induced an accumulation of ABA in strawberry receptacle tissue, but it remains unclear whether endogenous ethylene acts to promote ABA accumulation [80].

Strawberry has commonly been regarded as a typical NC fruit, and as such its ripening mechanism should be representative of canonical NC fruits. However, given the fact that strawberry fruit ripening is regulated by hormonal communication between the achenes and the receptacle, and the fact that not every NC fruit contains achenes, attention should be paid to potential diversity in the mechanism of hormonal regulation. Past studies have been largely based on pharmacological experiments, transient expression or heterologous expression. To conclusively demonstrate the role of hormones, manipulation of hormone metabolism or signaling via stably transgenic strategies will be necessary.

### Environmental signals regulating fruit ripening

Plants have evolved many strategies to cope with environmental stresses. While major attention has been paid to effects of environmental stress signals on plant vegetative growth and development, there have been far fewer studies on the responses of reproductive organs (i.e. flower and fruits). Fruits function in the dispersal of seeds and to promote reproductive success in an ever-changing environment [1, 15, 81]. From an evolutionary perspective, the reproductive response may represent a valuable strategy for coping with environmental stresses, as it acts to enable modulation of the mode of seed dispersal. Indeed, many plants survive extremely harsh environments, such as desert and alpine regions, due to their short life cycles [82]. Given that fruit ripening is such an important part of the life cycle of many plants, it is not surprising that many studies have shown that the progression of fruit ripening is sensitive to environmental signals. For example, the progression of strawberry fruit ripening is extremely sensitive to illumination, temperature and water availability [17, 83]. A minor elevation in temperature, (e.g. a few degrees) can shorten ripening by several days, which represents a far greater effect that any hormonal application. Environmental signals not only modulate fruit ripening, but also strongly affect fruit quality, which is particularly important in horticultural production [84].

Given the importance of environmental signals for both fruit ripening and quality, there is great interest in uncovering the underlying signaling mechanisms [85]. Such data can provide valuable guidance for investigating fruit ripening and quality formation in response to environmental signals. Studies of the effects of environmental factors on fruit ripening and quality have mainly focused on physiological and biochemical aspects, and relatively little is known about the associated signal transduction systems. Recently, Mao *et al.* (2022), reported that low temperature-inhibited anthocyanin accumulation in strawberry fruit is mediated by a FvMAPK3/FvMYB10 signaling module [86], which provided a clue for further elucidating the signaling mechanisms. While calcium-dependent protein kinase (CDPK) and mitogen activated protein kinase (MAPK) have been well established to be key signaling components in stress signaling, the report by Mao *et al.* (2022) provided direct evidence for the role of MAPK in strawberry fruit ripening and quality formation in response to a low temperature signal.

As described above, ABA has been suggested to be an important signal in NC fruit ripening. As ABA biosynthesis can be induced by salt stress, water deficit, and other environmental stresses [18, 87, 88], it may be that the responses of fruit ripening to environmental signals involve the ABA signaling pathway. The environmental signals may also modulate other hormonal signaling networks, as the metabolism and signaling of many hormones are affected by environmental stresses. Given that ABA has been well established to play an important role in NC fruit ripening and its biosynthesis is induced by environmental stresses, an important focus may be on the identification of the signaling pathway mediating the ABA signal in NC fruits. As mentioned above, ABA biosynthesis is determined by NCED, and we recently demonstrated that ABA signal production in strawberry fruit is mainly controlled by *NCED* gene expression. Accordingly, identification of the TFs controlling *NCED* expression as well as the upstream signaling components may be key in understanding environmental signal-modulated NC fruit ripening and quality attributes.

**Figure 1:**
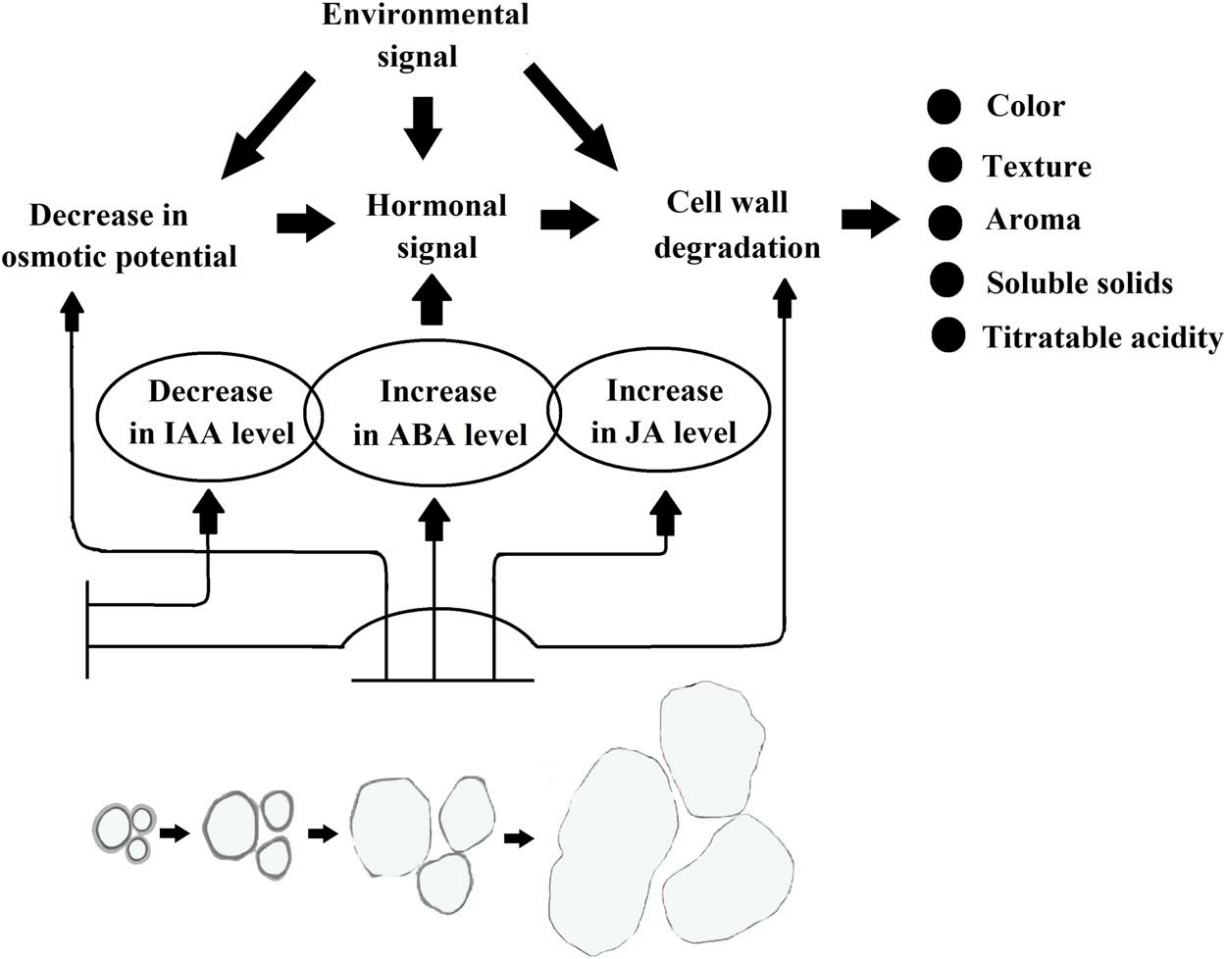
Primary signals implicated in the regulation of non-climacteric (NC) fruit ripening. NC fruit ripening is regulated by both internal and external cues, e.g. plant hormones and environmental signals, respectively. During fruit growth and development, there is a decrease of osmotic potential resulting from the accumulation of soluble solids and intercellular separation resulting from degradation of the cell wall and middle lamella. Cell separation can start from the very early stage of fruit set, and as such the two events may potentially serve as early signals, contributing to the initiation of the hormone signals and their signal amplification. Primary signals trigger cellular signal transduction, thereby controlling the changing pattern of fruit ripening-associated metabolism.

As summarized in Fig. 1, NC fruit ripening is regulated by both hormone signals and environmental signals. Among the plant hormones, IAA acts as a negative signal, a decline in which contributes to delaying fruit ripening, whereas ABA acts as a positive signal that promotes ripening. JA may also serve as a positive signal, possibly at later ripening stages. Moreover, a decrease in osmotic potential resulting from soluble solid accumulation may function as an early signal that contributes to the initial ABA signal or to independently promote fruit ripening. Cell separation and wall degradation have been proposed to be additional factors that regulate NC fruit ripening. In this model, degradation of the cell wall initiates overall fruit ripening, and therefore contributes to an amplification of the hormone signals. Environmental signals play an important role in both fruit ripening and quality, likely via modification of hormone signaling or by independently modifying fruit ripening or quality. Taken together, there is growing evidence that NC fruit ripening is regulated by the synergistic action of multiple signals.

### TFs regulating fruit ripening

TFs act to directly regulate the expression of genes involved in essentially all metabolic processes and so the identification and characterization of TFs controlling fruit ripening is key to deciphering the complex fruit ripening signaling network. In the past years, many TFs have been identified to be involved in the regulation of strawberry fruit ripening. As changes in strawberry fruit color are a readily recognizable event during ripening, most of the TFs identified to date are involved in the regulation of anthocyanin pigment accumulation, and hence red coloration. MYB TFs, in particular MYB10, are known to be critical for controlling fruit color [89–96]. MYB10 binds to the promoter region of key anthocyanin biosynthesis genes, thereby activating their expression [95]. In support of the finding that FaMYB10 plays a major role in controlling fruit color [89, 91–93, 95], the FaRAV1 TF, which is related to the abscisic acid insensitive 3 (ABI3)/viviparous TF, was reported to regulate anthocyanin production by bindings to the FaMYB10 promoter and activating its expression [97]. Notably, in addition to regulating anthocyanin production, FaMYB10 was also reported to be involved in sucrose metabolism via an interplay with FaMYB44.2 [98].

The regulation of anthocyanin production is complex. In addition to FaMYB10, many other TFs have been reported to play roles in anthocyanin production in both strawberry and other NC fruits [99–101]. Recently, FvTCP9, TEOSINTE BRANCHED 1, CYCLOIDEA, and PROLIFERATING4 CELL FACTORS were reported to physically interact with FaMYC1 to modulate anthocyanin biosynthesis [99], and another study demonstrated that a bHLH transcription factor enhances anthocyanin biosynthesis by specifically binding to the promoter region of key genes, including *FvDFR* (dihydroflavonol 4-reductase gene), and forming a HY5-bHLH9 transcription complex in strawberry fruit [101]. More recently, a potential role for AP2/ERF (APETALA2/ethylene-responsive element-binding factor) in both color and aroma formation in strawberry fruit was suggested by gene expression profile analysis [100].

In grape berry, several studies have demonstrated the involvement of different MYB TFs in fruit color regulation [94, 102–105]. An early study demonstrated that transient overexpression of the grapevine TF VvMYBPA1 activated the promoter of several genes involved in the proanthocyanidin biosynthesis pathway, including those encoding leucoanthocyanidin reductase (LAR) and anthocyanidin reductase (ANR) [105], and ectopic expression of the VvMYB5b grape TF in tobacco plants induced anthocyanin and proanthocyanidin accumulation [94]. In contrast, transient overexpression of VvMYBC2-L1in grape hairy roots induced substantial reductions in the expression of many genes in the proanthocyanidin pathway, such as VvDFR (dihydroflavonol reductase), VvLDOX (leucoanthocyanidin dioxygenase), VvLAR1 and VvLAR2 (leucoanthocyanidin reductase), and VvLAR2 (anthocyanidin reductase), suggesting that VvMYBC2 is a negative regulator [103]*.* More recently, it was reported that overexpression of VviMYB86 promoted LAR expression, whereas expression of VviANS and VviUFGT, two key genes involved in grape callus anthocyanin biosynthesis [104], and ectopic expression of VvMYBA2 in tobacco caused an up-regulation of anthocyanin biosynthetic genes and resulted in higher anthocyanin accumulation [102].

In addition to the TFs involved in fruit color regulation, there is evidence that MYB TFs are also involved in the regulation of other metabolic processes [98, 106, 107]. For example, FaMYB44.2 from strawberry functions in the regulation of sucrose accumulation through an interplay with FaMYB10. Additionally, an R2R3-MYB TF regulates the synthesis of the aromatic compound eugenol in ripe strawberry fruit receptacles [98] and an ETHYLENE RESPONSE FACTOR-MYB transcription complex regulates the biosynthesis another aroma compound, furaneol [106]. In grape berry, the R2R3-MYB, VvMYBF1, was demonstrated to regulate flavonol synthesis [108], and VdMYB1 and VviMYB13, two other R2R3-MYB TFs, were reported to be involved in stilbene accumulation [109, 110]. In addition, a variety of other TF families have been demonstrated to play roles in regulating many other fruit ripening associated-metabolic processes [90, 111–115].

Genome-wide analysis of the NAC TF family suggested that the expression profiles of many members were tightly associated with strawberry fruit development and ripening [116]. Promoter analysis using a dual luciferase technique demonstrated that FcNAC1 from *Fragaria chiloensis* binds to the promoter of a pectate lyase gene involved in the depolymerization cell wall pectin, thereby linking FcNAC1 with ripening-associated textural changes [114]. More recently, it was demonstrated that down-regulation of FaRIF, a NAC TF, via RNAi mediated silencing caused delayed strawberry fruit ripening. Functional identification of TFs is commonly conducted by transient or ectopic gene expression due to the technical difficulties of stably manipulating gene expression in strawberry; however, this study provided direct evidence that NAC TFs play roles in the regulation of strawberry fruit ripening [112].

There is evidence that TFs from many other families having a role in strawberry fruit ripening. For example, using fruit transient expression, it was demonstrated that FaSHP, a C-type MADS-box SHATTERPROOF-like TF, was important [113], and the fruit-specific FaDOF2 TF, a member of the plant-specific Dof (DNA binding with one finger) family, which is known to be involved in growth, development, and stress responses [117], was shown to be involved in the regulation of eugenol production in strawberry receptacles [111]. In grape berry, population genetics and transgenic analysis suggested that VvNAC26 and VvCEB1 are involved in the regulation of berry size [118] and shape variation [119], respectively. It has also been shown that stably overexpressing VviERF045, a berry-specific ethylene responsive factor, affected cuticular waxes and phenolic compound metabolism [120]. Finally, a number of studies have implicated WRKY TFs in fruit ripening-associated metabolic pathways, such as flavonoid biosynthesis [115], monoterpenoid production [101], and sugar accumulation [121].

### The signaling relay from primary signal perception to signaling output

Signal transduction consists of the initial signal perception, an intermediate signaling relay and a final signaling output. The intermediate signaling relay can be achieved by different mechanisms, such as reversible protein phosphorylation, ubiquitylation, SUMOylation, neddylation, glycosylation, and acetylation. Signal in CL fruits transduction has been extensively studied and reviewed, particularly focusing on ethylene signaling in tomato [3–7, 13–16]. However, far less is known about the equivalent intermediate signaling relay systems NC fruits.

In this regard, given that ABA is considered to be an important signal controlling NC fruit ripening, ABA has received the most attention. Several studies have reported that manipulating the genes encoding signaling components, such as the ABA receptor, FaPYR1 [51, 57], the co-receptor, FaABI1 [56], and FaSnRK2.6 [122], a central component in the ABA signaling pathway, caused a modification of the ripening progress in strawberry fruit. Notably, strawberry ripening involves multiple other hormones and so many other signaling cascades may be involved. One study reported that strawberry fruit ripening was promoted by sucrose treatment and over-expression/RNAi down-regulation of *FaSUT1,* a sucrose transporter, resulted in a promotion and depression of fruit ripening, respectively, indicating that sucrose might serve as a signal controlling ripening [123]. In addition, our studies demonstrated that a FERONIA-like receptor kinase, FaMRLK47, was involved in the regulation of strawberry fruit ripening [124]. FaMRLK47 physically interacts with FaABI1, and its overexpression or RNAi-mediated downregulation was found to cause a decrease or increase, respectively, in the ABA-induced expression of a series of ripening-related genes [125].

Transcriptome studies of strawberry fruit have revealed many of the signaling components whose expression profile correlates with ripening. For example, the expression of three annexin genes, encoding calcium-binding proteins, was found to be closely correlated with ripening [36], as was the expression of several calcium-dependent protein kinases (CDPKs) [126, 127], mitogen-activated protein kinases (MAPKs) and malectin-like domain-containing receptor-like kinase (MRLK) genes [128, 129]. RNA-based signals are involved in the regulatory system; for example, in strawberry a miRNA, Fan-miR73, was implicated in the regulation of ripening via targeting of ABI5 [130], and a noncoding RNA, FRILAIR, can act as a noncanonical target mimic of miR397 to modulate the ripening process [131].

Compared with strawberry, there are fewer studies of ripening-associated signal transduction in grape berry. One study showed that heterologous expression of VlPYL1, a putative ABA receptor, in Arabidopsis enhanced ABA sensitivity and that transient over-expression of *VlPYL1* in grape berry promoted anthocyanin accumulation [132]. Moreover, individually silencing the hexokinases CsHXK1 or CsHXK2, which are thought to function as glucose sensors, in grape calli reduced the expression of sucrose synthase and cell wall invertase-encoding genes, suggesting an involvement of sugar signaling in the regulation of grape berry ripening [133]. The serine/threonine kinase, glycogen synthase kinase 3/shaggy kinase (GSK3) is involved in brassinosteroid signaling, and one study reported that transient overexpression of the grape homolog, VvSK, in tomato delayed ripening [134]. It has also been reported that a U-Box E3 Ubiquitin ligase U-box (PUB) gene, *VIPUB35*, is involved in grape fruit ripening, and its heterologous expression in strawberry delayed ripening. VlPUB35 interacts with abscisic-aldehyde oxidase (VlAAO), an enzyme involved in ABA catabolism, thereby promoting its degradation, suggesting that the ripening modulation by VIPUB35 operates via regulation of ABA levels [135].

**Figure 2:**
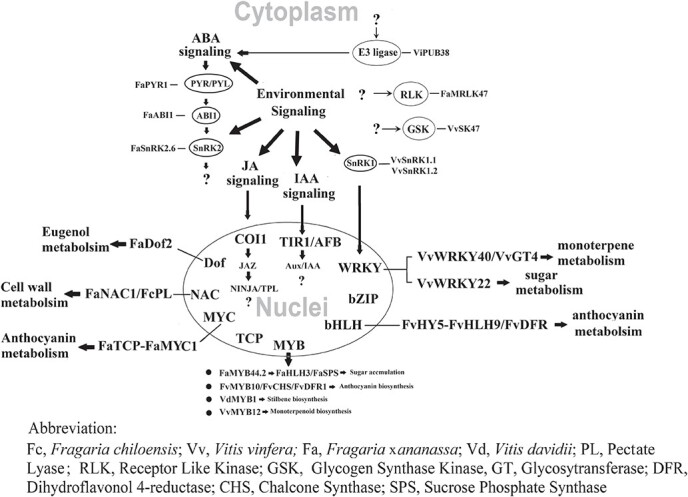
Fruit ripening-associated signal transduction. Perception of the primary internal signals (IAA, ABA, and JA) as well as environmental signals act to initiate an intermediate signaling relay, which leads to regulation of downstream transcription factors (TFs), and finally, the regulation of a diversity of ripening-associated metabolic pathways. Circled are the types of signaling components TFs implicated in the regulation of non-climacteric (NC) fruit ripening. The names within the ovals refer to the specific signaling components or TFs that have already been identified and characterized in strawberry or grape berry. Question marks represent putative events that have not yet been characterized.

Although previous studies have shed much light on primary signals and TFs, less is known about the intermediate signaling relay. As mentioned above, the intermediate signaling relay can be achieved by a diversity of modification mechanisms of the signal proteins, of which protein kinase/phosphatase-catalyzed reversible protein phosphorylation represents the major mechanism. The protein kinase superfamily comprises many subfamilies, each of which contains many protein kinases. For example, the receptor-like protein kinase (RLKs) subfamily contains more than 600 members in Arabidopsis [136]. However, little is known about their roles in fruit ripening-associated signal transduction.

To date, most studies of signal transduction in NC fruit ripening have been based on two techniques: (i) gene expression analysis to examine whether the expression profiles of certain genes correlate with ripening; and (ii) functional identification via transgenic manipulation, including transient or stable expression. Due to the difficulty of stably expressing transgenes in grape, nearly all grape gene-related studies are based on transient or heterologous expression, and although this may provide important information, these techniques may have some adverse effects on fruit ripening, causing unreliable results or confounding conclusions.

## Conclusion and perspective

Fruit ripening is controlled by a complex signaling network consisting of many intertwined signaling cascades (Fig. 2). Phytohormones represent primary signals, and play key roles in fruit ripening. In CL fruits, ethylene is well established as the major hormone controlling fruit ripening. However, many hormones have been suggested to be involved in the regulation of NC fruit ripening, and there is no conclusive evidence that there are specific predominant hormones. Rather, it is likely that NC fruit ripening is controlled by the synergistic action of multiple hormones rather than a single hormone acting on its own. It is also important to realize that while plant hormones have generally been regarded as primary signals, attention should be paid to earlier signals that may modulate the origin of the hormone signal, such as cell separation-associated signals, osmotic potential change-associated signals, etc. Environmental signals also act as primary signals, and play pivotal roles in both fruit ripening and quality formation. Finally, as the terminal signal of the primary signal-initiated signaling cascade, TFs regulate the gene expression of the proteins that directly control the metabolic basis of fruit ripening. In recent years, while an increasing number of TFs have been identified, far less is known about their regulation. To elucidate the mechanisms for NC fruit ripening, we propose several potential research targets:

### Identification and characterization of key hormones controlling NC fruit ripening

As CL ripening is well known to be controlled by a major hormone (ethylene), identification of the corresponding key hormone in NC fruit ripening has been of major interest. Particular attention has been paid to the role of ABA in NC fruit ripening; however, it does not appear that ABA is the predominant hormone controlling this process, and many other phytohormones, such as IAA and JA, have been also shown to contribute. Moreover, there are reports that the direct introduction of ABA into the strawberry receptacle does not have a measurable effect on fruit ripening [48, 60]. Given that strawberry fruit ripening has been demonstrated to be controlled by a synergistic action between IAA and ABA [60], it is important to better understand their synergistic relationships in other NC fruits, and whether this represents a major mechanism for ripening regulation.

### Mechanistic investigation of hormone signal origins

The origin of hormonal signals (i.e. increase or decrease in the level of the hormone at the onset of fruit ripening) is a programed process that may be triggered by other earlier signals. Decreased osmotic potential as a result of soluble solid accumulation might be provide such a signal in strawberry fruit ripening, and because ABA accumulation can be induced by a decreased osmotic potential [85], it could be interesting to investigate whether the origination of the ABA signal correlates with decreased osmotic potential. More importantly, ethylene signal production is essentially a process of autocatalytic ethylene biosynthesis (i.e. a small amount of initial ethylene is capable of triggering ethylene production in large quantities [7, 137, 138]) and we recently demonstrated that ABA accumulation in the achenes is also an autocatalytic biosynthetic process [60]. Because ABA biosynthesis is controlled by NCED, identification of the signaling components or pathway involved in NCED regulation may be central to mechanistic studies regarding the origin of the ABA signal.

### Mechanistic study of environmental signal-modulated NC fruit ripening and quality formation

Both fruit ripening and quality can be strongly modulated by a variety of environmental signals, such as light, temperature, water, and salt stress. Studies of environmental signaling have mainly focused on plant stress adaptation, and little is known about environmental signal-modulated NC fruit ripening and quality formation. Knowledge of environmental signaling in relation to plant stress adaptation may give important insights into NC fruit ripening and quality.

### Identification of the signaling components and pathways upstream from key TFs controlling NC fruit ripening

As mentioned above, molecular studies of fruit ripening have mainly focused on identification of TFs, of which many have been found and demonstrated to play pivotal roles in NC fruit ripening. Identification of the signal components or pathways upstream of these TFs is essential to gain a deep understanding of the molecular mechanisms underlying NC fruit ripening. Comprehensive identification and characterization of the proteins that physically interact with the TFs will likely be extremely important in elucidating the associated signaling networks and key regulatory points.

### Establishment of technical resources for studies of signal transduction during NC fruit ripening

The relatively slow progress in characterizing NC fruit ripening-related signal transduction has largely been due to the lack of well-established systems for molecular manipulation, such as stable transgene expression and screening of mutant populations. However, there has been recent progress with strawberry in establishing stable transgenic lines, as well as in the application of clustered regularly interspaced short palindromic repeats (CRISPR/Cas9) technology [139]. To accelerate the understanding of the molecular mechanisms underlying ripening in NC fruit, and the associated signal transduction networks, similar technologies and resources should be developed for a wide range of NC fruit species.
